# Supplementary material for: CircANKRD12 Is Induced in Endothelial Cell Response to Oxidative Stress
Source: Cells. 2022 Nov 9;11(22):3546. doi: 10.3390/cells11223546 (PMC9688326; doi:10.3390/cells11223546)
Supplement: Supplementary file 1 [file cells-11-03546-s001.zip › Supplementary.Material_Voellenkle/Supplementaries_Voellenkle_proofs.pdf]

CircANKRD12 is induced in endothelial cells response to oxidative stress

Christine Voellenkle, Paola Fuschi, Martina Mutoli, Matteo Carrara, Paolo Righini, Giovanni Nano, Carlo Gaetano, Fabio Martelli

Supplementary Material

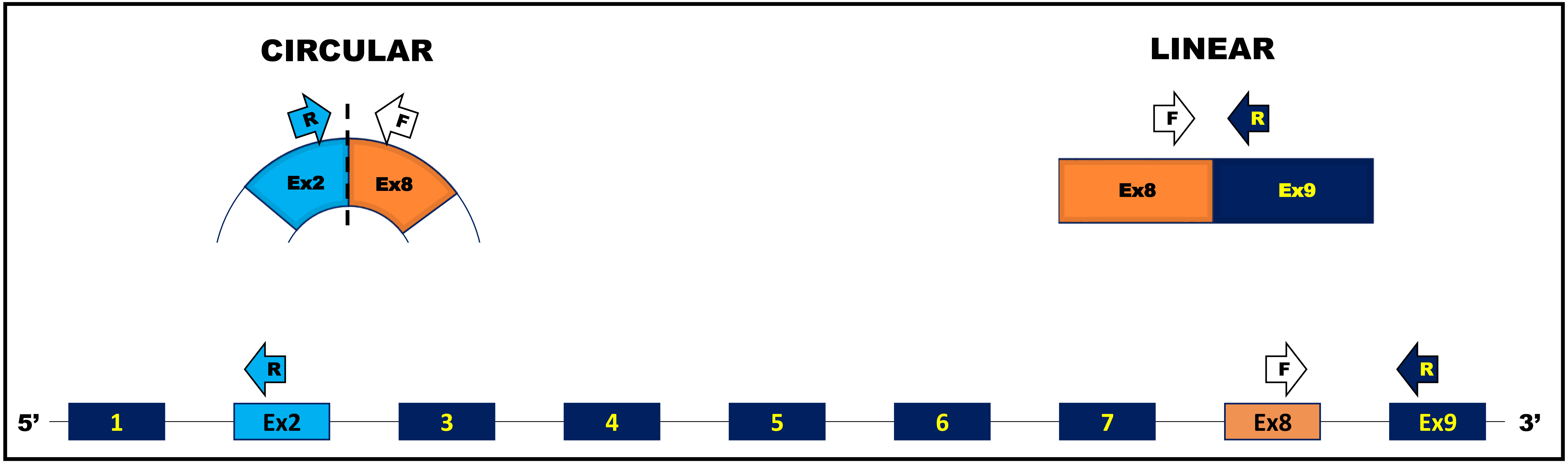

Figure S1. Design of primers for candidate transcripts. ANKRD12 is shown as example of the primer-design used for the detection of circular (divergent primers) and linear (convergent primers) transcripts, sharing the same forward primer. The broken line indicates the back-splice junction. Underneath, a schematic representation of primer locations on the pre-mRNA.

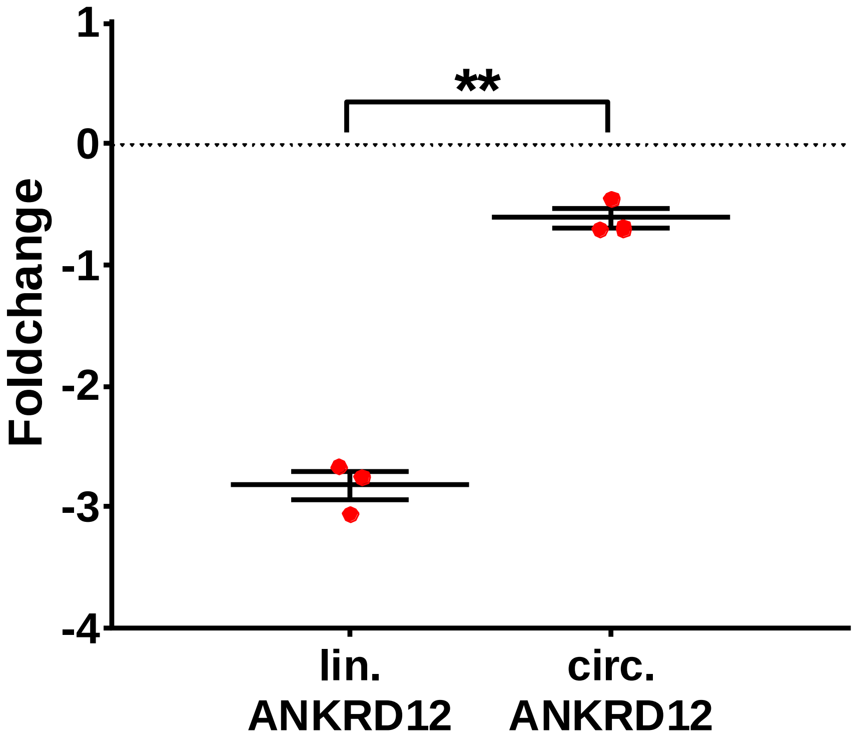

Figure S2. Resistance of circular ANKRD12 to exoribonuclease R. HUVEC-derived total RNA was incubated with or without RNase R. Expression of linear and circular transcripts was measured by qPCR. Scatterplot shows fold changes representing the differences in abundance of RNase R digested compared to non-digested for each transcript type (n=3); Fold changes are in log2 scale (-ΔΔCT) and individual values are indicated by dots, while mean and SEM are indicated by horizontal lines (n=3; **$p < 0.01$).

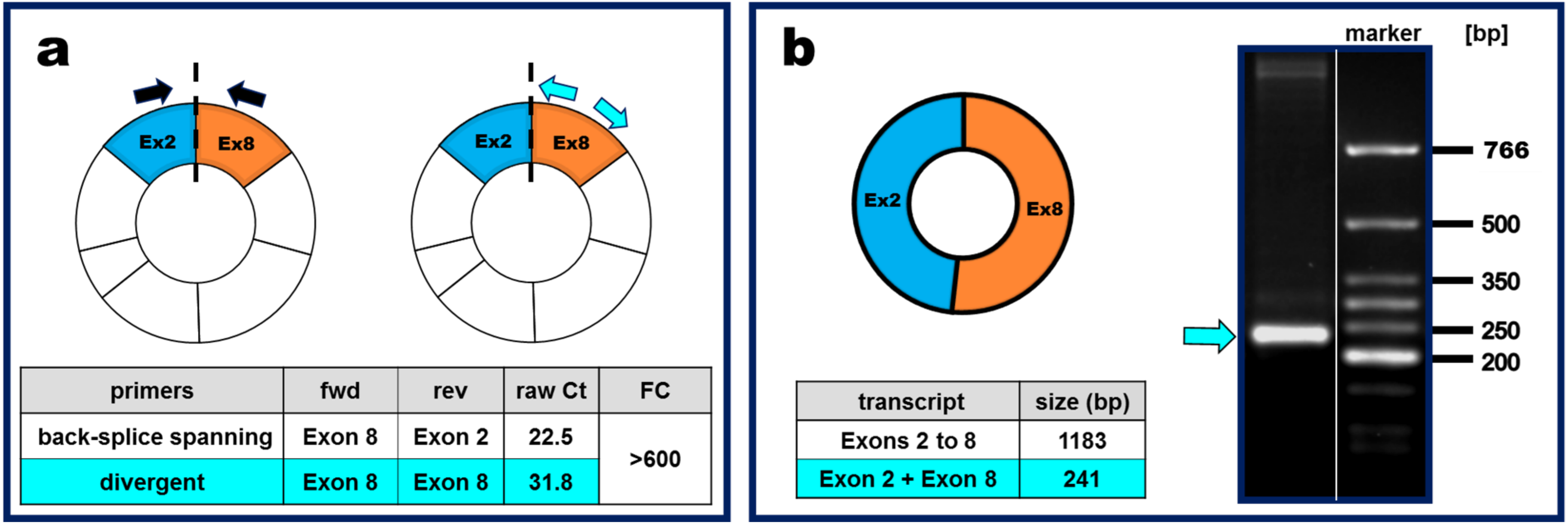

Figure S3. Identification of the short circANKRD12 isoform in human endothelial cells. Investigation of an additional isoform comprised of only two exons (Exon 2 and 8). **(a)** Table showing details of the used primer pairs, all displaying similar efficiencies, together with their expression levels as estimated by qPCR. Although no accurate measurement was performed, data indicate low levels of the short circANKRD12 isoform compared to the longer isoforms. Drawings show the orientation of the primers on the circular transcripts. The black arrows represent the primers spanning the back-splice junction of all circANKRD12 isoforms, the turquoise arrows the divergent primers on Exon 8. The broken line indicates the back-splice junction. **(b)** Agarose gel analysis performed with amplicons produced by divergent primers on Exon 8. The arrow indicates a band at the size expected for the short isoform, depicted above.

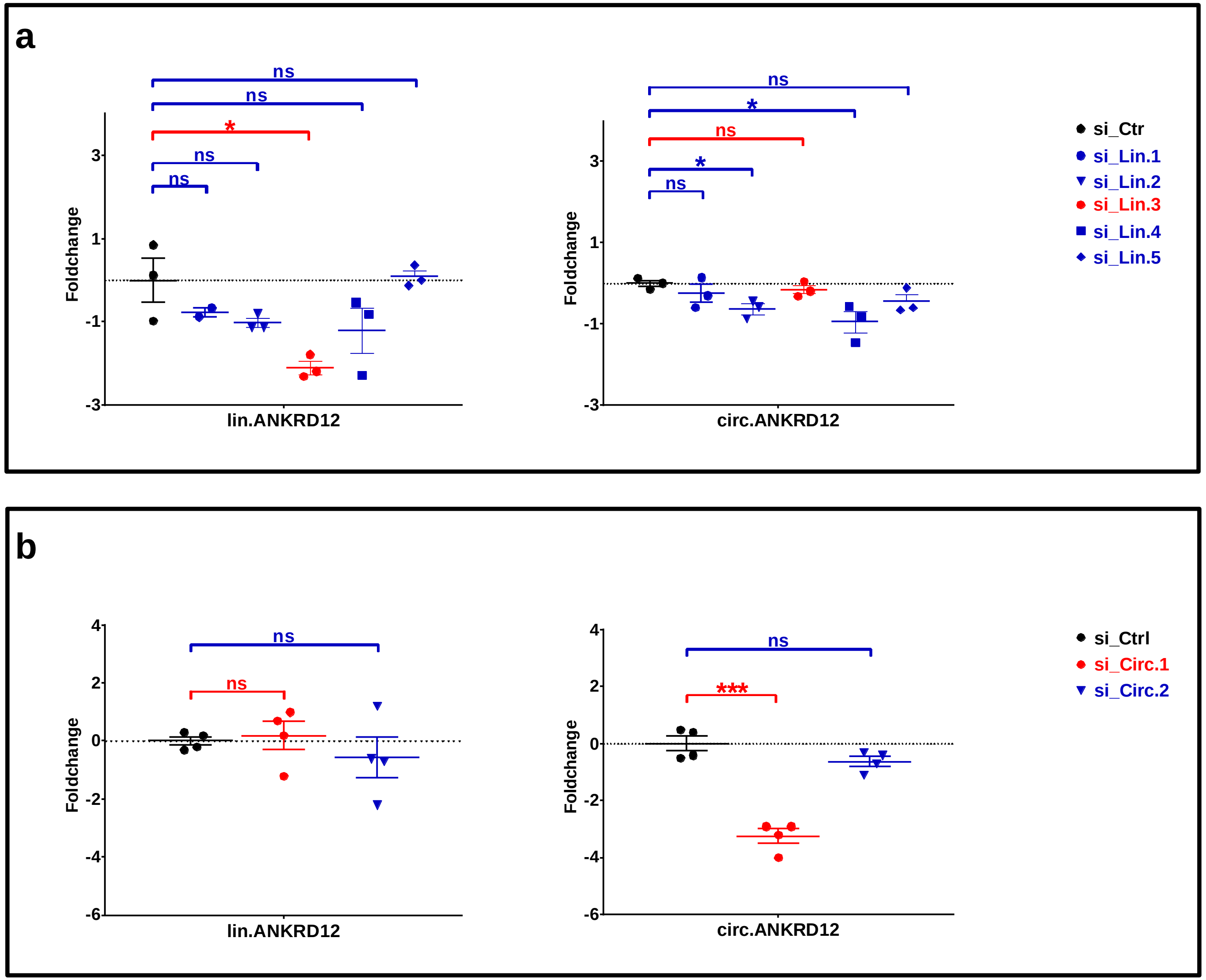

Figure S4. Screening for efficient and transcript-specific knockdown of ANKRD12 in HUVEC. siRNAs designed for knockdown of either **(a)** the linear (si_Lin.) or **(b)** the circular (si_Circ.) transcripts of ANKRD12 were analyzed by qPCR. As negative control, a non-targeting siRNA (si_Ctrl) was used. Scatterplots show the expression levels of linear and circular ANKRD12 RNAs following knockdown. Efficient siRNAs chosen for all subsequent functional studies are highlighted in red. Fold changes are in log2 scale (-ΔΔCT) and individual values are indicated by dots, while mean and SEM are indicated by horizontal lines (n=3, $*p < 0.05$, $***p < 0.001$; ns: not significant).

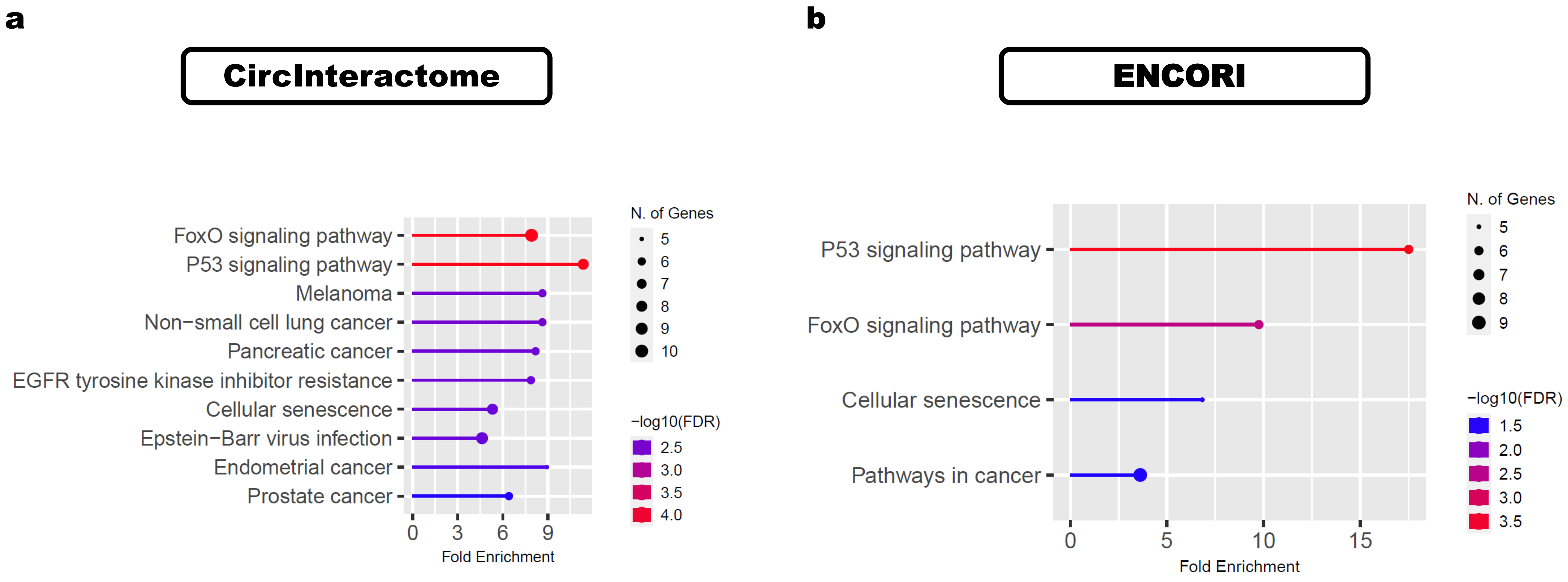

Figure S5. Top enriched KEGG-pathways predicted for circANKRD12 – miRNA – mRNA networks. Lollipop charts sorted by FDR of **(a)** the top 10 enriched pathways resulting from the CircInteractome-pipeline and **(b)** pathways showing significant enrichment using the ENCORI-pipeline.

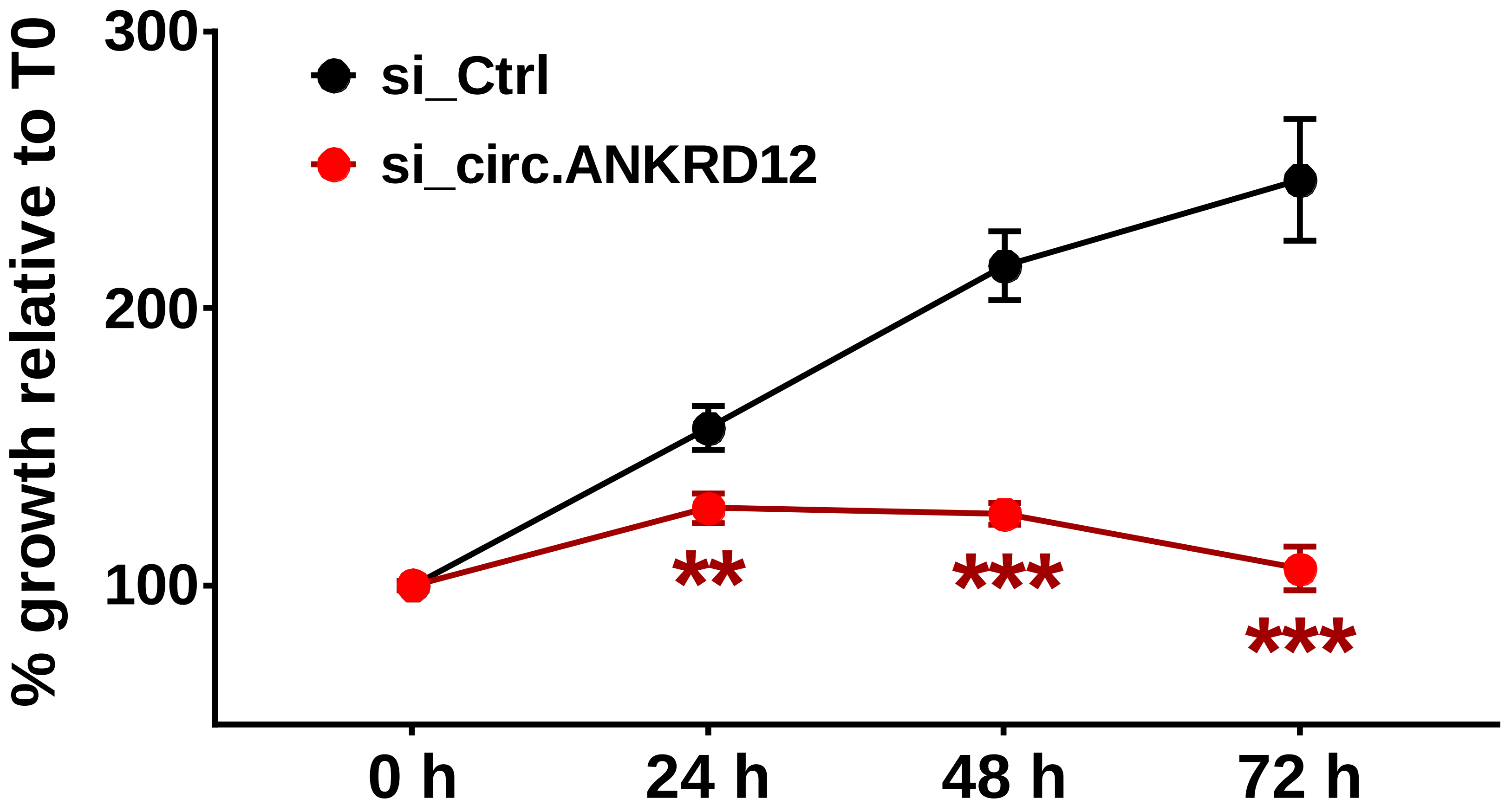

Figure S6. Knockdown of circular ANKRD12 leads to reduced HUVEC proliferation. HUVEC were transfected with siRNA specific for the back-splice junction of circ.ANKRD12. As negative control a non-targeting siRNA (si_Ctrl) was used. Cell growth was monitored by measuring absorbance of crystal violet. Two independent proliferation assays (each n=12) were aggregated by averaging absorbance-values expressed as percentages relative to time point 0, (n=24; **$p < 0.01$, ***$p < 0.001$).
